# Prognostic significance of sarcopenia diagnosed based on the anthropometric equation for progression-free survival and overall survival in patients with colorectal cancer

**DOI:** 10.3389/fnut.2023.1076589

**Published:** 2023-02-01

**Authors:** Hailun Xie, Lishuang Wei, Shunhui Gao, Mingxiang Liu, Yanren Liang, Guanghui Yuan, Qiwen Wang, Yansong Xu, Shuangyi Tang, Jialiang Gan

**Affiliations:** ^1^Department of Colorectal and Anal Surgery, The First Affiliated Hospital, Guangxi Medical University, Nanning, Guangxi, China; ^2^Guangxi Key Laboratory of Enhanced Recovery After Surgery for Gastrointestinal Cancer, The First Affiliated Hospital, Guangxi Medical University, Nanning, Guangxi, China; ^3^Department of Geriatric Respiratory Disease Ward, The First Affiliated Hospital, Guangxi Medical University, Nanning, Guangxi, China; ^4^Department of Emergency Surgery, The First Affiliated Hospital, Guangxi Medical University, Nanning, Guangxi, China; ^5^Department of Pharmacy, The First Affiliated Hospital, Guangxi Medical University, Nanning, Guangxi, China

**Keywords:** sarcopenia, malnutrition, progression-free survival, overall survival, colorectal cancer

## Abstract

**Background:**

The purpose of this study was to investigate the prognostic significance of sarcopenia diagnosed based on anthropometric equations for progression-free survival (PFS) and overall survival (OS) in patients with colorectal cancer (CRC).

**Methods:**

A total of 1,441 CRC patients who underwent surgical treatment between January 2012 and December 2016 were enrolled in this study. Sarcopenia was diagnosed according to validated anthropometric equations. The Kaplan–Meier method with the log-rank test was used to estimate the survival curve. Cox proportional hazards regression models with forward selection were used to evaluate risk factors affecting the prognosis of CRC patients. R package “survival” was used to build the prognostic nomograms to predict 1–5 years of PFS and OS in CRC patients. The concordance index (C-index) and calibration curve were used to evaluate the prognostic accuracy of the prognostic nomogram.

**Results:**

Two hundred and seventy-one patients (18.8%) were diagnosed with sarcopenia. Sarcopenia was significantly associated with advanced age, large tumor size, and high mortality. Compared with the non-sarcopenia patients, the PFS of sarcopenia patients was worse (5-year PFS, 48.34 vs. 58.80%, *p* = 0.003). Multivariate survival analysis showed that patients with sarcopenia had a higher risk (23.9%) of adverse PFS (HR, 1.239; 95%CI: 1.019–1.505, *p* = 0.031) than patients without sarcopenia. The OS of patients with sarcopenia was significantly worse than that of patients without sarcopenia (5-year OS: 50.92 vs. 61.62%, *p* = 0.001). In CRC patients, sarcopenia was independently associated with poor OS (HR: 1.273, 95%CI: 1.042–1.556, *p* < 0.001). Moreover, sarcopenia effectively differentiated the OS of CRC patients in the normal carcinoembryonic antigen (CEA) subgroup but not in the high CEA subgroup. Notably, sarcopenia can provide effective prognostic stratification in CRC patients at different pathological stages. Nomograms that integrated prognostic features were built to predict the risk of adverse outcomes in CRC patients. The C-index and calibration curves showed that these nomograms had good prediction accuracy. Internal validation confirmed that our nomogram has wide application potential.

**Conclusion:**

Sarcopenia diagnosed based on anthropometric equations is an independent risk factor for PFS and OS in CRC patients.

## 1. Introduction

Colorectal cancer (CRC) is a common malignancy of the digestive system. Previous reports have stated that CRC is the third most common cancer in men and the second most common cancer in women, accounting for 10.6 and 9.4%, respectively. Furthermore, CRC is the third leading cause of cancer-related death in men and women, accounting for more than 510,000 deaths in men and more than 420,000 deaths in women (1). In China, the incidence and mortality of CRC are on the rise. The incidence and mortality of CRC ranked second and fifth among all malignancies, respectively (2). Therefore, early identification of poor prognostic factors in CRC patients and timely interventions are urgently needed to improve the prognosis in these patients.

CRC is often accompanied by low food intake, malabsorption of nutrients, and high systemic inflammation, which transform the body into a state of high catabolic and low synthetic malnutrition. Particularly, these patients often present with progressive loss of body mass, including reduced muscle mass, strength, and muscle function, leading to sarcopenia and even cachexia. Sarcopenia significantly affects the prognosis of patients with malignancies. It is a well-known independent risk factor for poor prognosis in malignancies (3–6). There is a 12–60% prevalence of sarcopenia in CRC patients (7, 8). Early detection of sarcopenia and maintenance of skeletal muscle mass and function are important goals for diagnosing and treating cancer patients. The main methods to diagnose sarcopenia include Dual Energy X-Ray Absorptiometry (DXA), Computed Tomography (CT), Magnetic Resonance Imaging (MRI), and Bioelectrical Impedance Analysis (BIA) (9–11). These diagnostic methods are expensive, time-consuming, cumbersome, or radioactive, so they are poorly used in clinical practice. It is, therefore, necessary to develop a simple, convenient, and reliable diagnostic method to diagnose sarcopenia.

Recently, Wen et al. (12) developed an anthropometric equation for appendicular skeletal muscle (ASM) based on the Chinese population. It is a simple and practical diagnostic tool for sarcopenia. The accuracy of this tool was verified in several studies (13–15). However, there are few studies focused on the correlation between using this equation to diagnose sarcopenia and the prognosis of CRC patients. The purpose of this study was to investigate the prognostic value of sarcopenia diagnosed based on the anthropometric equation in progression-free survival (PFS) and the overall survival (OS) of CRC patients. Therefore, this study aims to become a reference point for the clinical diagnosis, treatment, and research of sarcopenia in CRC patients.

## 2. Patients and methods

### 2.1. Study population and data collection

This study included CRC patients who underwent surgical treatment in the Department of Colorectal and Anal Surgery, the First Affiliated Hospital of Guangxi Medical University, between January 2012 and December 2016. The inclusion criteria were as follows: (1) pathological analysis confirmed that the primary lesion was CRC; (2) patients who had not received preoperative neoadjuvant chemoradiotherapy; and (3) patients who had undergone surgical resection for CRC. The exclusion criteria were as follows: (1) patients with unclear primary tumor sites or multi-site tumors; (2) patients < 18 years old; and (3) patients who refused to participate in this study. This study was conducted in strict accordance with the provisions of the Declaration of Helsinki. In addition, written informed consent was obtained from all patients or their close relatives. The Ethics Review Board of the First Affiliated Hospital of Guangxi Medical University approved this study, with the approval number: 2021 (KY-E-043).

Clinicopathological information of CRC patients was retrospectively collected. The information included sex, age, height, weight, body mass index (BMI), history of hypertension, diabetes, serum carcinoembryonic antigen (CEA) level, *T* stage, *N* stage, preoperative metastasis, tumor-node-metastasis (TNM) stage, perineural invasion, vascular invasion, pathological type, differentiation, tumor location, tumor size, surgical approach, postoperative radiotherapy, and postoperative chemotherapy.

### 2.2. Diagnosis of sarcopenia

Sarcopenia is a syndrome primarily characterized by a progressive, generalized reduction in skeletal muscle mass and functional decline. In this study, we estimated the ASM based on the anthropometric equation, which was calculated as follows: 0.193 × weight (kg) + 0.107 × height (cm) −4.157 × sex (male = 1, female = 2) −0.037 × age (year) −2.631. The Skeletal Muscle Index (SMI) was defined as ASM/height squared (m^2^), that is, SMI = ASM/Ht squared (Kg/m^2^). The updated consensus on the diagnosis and treatment of sarcopenia published by the Asian Working Group on Sarcopenia (AWGS) in 2019 is widely used as the diagnostic criteria for sarcopenia in the Asian population. That is, SMI < 6.92 Kg/m^2^ in a man, and SMI < 5.13 Kg/m^2^ in a woman is considered sarcopenia (16).

### 2.3. Follow-up and outcomes

Patients who recovered well were discharged from the hospital. After this, regular visits and telephone follow-ups were performed in the outpatient or inpatient departments. Patients were followed up every 3–6 months in the first year and every 6–12 months from the second year until death. The primary purpose of follow-up was to record the patients' survival status, serum tumor markers, abdominal CT, and electronic fiber colonoscopy. PFS was defined as the interval between surgery and disease recurrence, death, or last follow-up. OS was defined as the interval between surgery and death from any cause or the last follow-up. The date of the last follow-up was February 4, 2021.

### 2.4. Statistical analysis

Data were expressed as mean ± standard deviation. An independent sample *t*-test was used to compare the measurement data, and the Chi-square test was used to compare categorical data. The Kaplan–Meier method was used to estimate the survival curve, and the log-rank test was used to compare survival rates. Cox proportional hazards regression models with forward selection were used to evaluate risk factors affecting the prognosis of CRC patients. R package “survival” was used to build the prognostic nomograms to predict 1–5 years of PFS and OS in CRC patients. The concordance index (C-index) and calibration curve were used to evaluate the prognostic accuracy of the prognostic nomograms. The total population was randomly divided into two internal validation datasets at a ratio of 7:3 to evaluate the practicability of the nomograms. Differences were considered statistically significant at two-sided *p*-values of <0.05. Statistical analyses were performed using the R software (Version 4.0.2).

## 3. Results

### 3.1. Clinicopathologic characteristics

A total of 1,441 CRC patients were enrolled in this study. According to the diagnostic criteria for sarcopenia, 271 patients (18.8%) were diagnosed with sarcopenia. Among the 904 enrolled male patients, 162 were diagnosed with sarcopenia, with an incidence of 17.9%. Among the 537 enrolled female patients, 109 were diagnosed with sarcopenia, with an incidence of 20.3%. The incidence of sarcopenia in female patients was slightly higher than that in male patients; however, there was no significant difference between the two groups (*p* = 0.295). The mean age of sarcopenia patients was 64.6 ± 13.4 years old, and the mean age of non-sarcopenia patients was 56.6 ± 12.6 years old. The mean age of patients with sarcopenia was significantly higher than that of patients without sarcopenia; there was a significant statistical difference between the two groups (*p* < 0.001). There were 705 patients with colon cancer, including 143 who were diagnosed with sarcopenia (20.3%), and 736 patients with rectal cancer, including 82 who were diagnosed with sarcopenia (17.4%). There was no significant difference in the incidence of sarcopenia among CRC patients with different tumor sites (*p* = 0.181). Interestingly, the tumor size in patients with sarcopenia was significantly larger than that in patients without sarcopenia (5.0 vs. 4.5 cm, *p* = 0.001). In addition, patients with sarcopenia also had a higher mortality risk than those without sarcopenia (49.1 vs. 38.4%, *p* = 0.002) (Table 1).

**Table 1:** The relationships between the sarcopenia and clinicopathological factors of CRC patients.

**Clinicopathological characteristics**	**Overall (*n* = 1,441)**	**Sarcopenia**		***P*-value**
		**No (*****n*** = **1,170)**	**Yes (*****n*** = **271)**	
Sex (Man)	904 (62.7)	742 (63.4)	162 (59.8)	0.295
Age [mean (SD)]	58.1 (13.2)	56.6 (12.6)	64.6 (13.4)	< 0.001
BMI [median (IQR)]	22.04 (19.95, 24.31)	22.84 (21.09, 24.98)	18.03 (17.02, 19.13)	< 0.001
Hypertension (Yes)	241 (16.7)	208 (17.8)	33 (12.2)	0.033
Diabetes (Yes)	90 (6.2)	77 (6.6)	13 (4.8)	0.34
***T*** **stage**				0.834
T1	50 (3.5)	39 (3.3)	11 (4.1)	
T2	318 (22.1)	262 (22.4)	56 (20.7)	
T3	770 (53.4)	621 (53.1)	149 (55.0)	
T4	303 (21.0)	248 (21.2)	55 (20.3)	
***N*** **stage**				0.831
N0	808 (56.1)	655 (56.0)	153 (56.5)	
N1	398 (27.6)	321 (27.4)	77 (28.4)	
N2	235 (16.3)	194 (16.6)	41 (15.1)	
*M* stage (Yes)	137 (9.5)	107 (9.1)	30 (11.1)	0.391
TNM stage (III–IV)	677 (47.0)	549 (46.9)	128 (47.2)	0.981
Perineural invasion (Yes)	149 (10.3)	126 (10.8)	23 (8.5)	0.317
Vascular invasion (Yes)	247 (17.1)	210 (17.9)	37 (13.7)	0.109
**Macroscopic type**				0.067
Protrude type	406 (28.2)	318 (27.2)	88 (32.5)	
Infiltrating type	113 (7.8)	87 (7.4)	26 (9.6)	
Ulcerative type	922 (64.0)	765 (65.4)	157 (57.9)	
Differentiation (Poor)	190 (13.2)	156 (13.3)	34 (12.5)	0.806
Tumor location (Rectal)	736 (51.1)	608 (52.0)	128 (47.2)	0.181
Tumor size [median (IQR)]	4.5 (3.5, 6.0)	4.5 (3.5, 6.0)	5.0 (3.6, 6.0)	0.001
CEA (High)	594 (41.2)	472 (40.3)	122 (45.0)	0.180
Surgical method (Open)	604 (41.9)	463 (39.6)	141 (52.0)	< 0.001
Radiotherapy (Yes)	134 (9.3)	120 (10.3)	14 (5.2)	0.013
Chemotherapy (Yes)	657 (45.6)	573 (49.0)	84 (31.0)	< 0.001
Death (Yes)	582 (40.4)	449 (38.4)	133 (49.1)	0.002
HOS [median (IQR)]	17.0 (11.0, 21.0)	17.0 (11.0, 21.0)	17.0 (12.0, 21.5)	0.077
Hospitalization cost [median (IQR)]	49,539.2 (44,565.3, 55,986.4)	49,399.3 (44,628.5, 55,673.4)	50,069.5 (44,036.4, 56,940.0)	0.519

### 3.2. The relationship between sarcopenia and PFS

**Figure 1:**
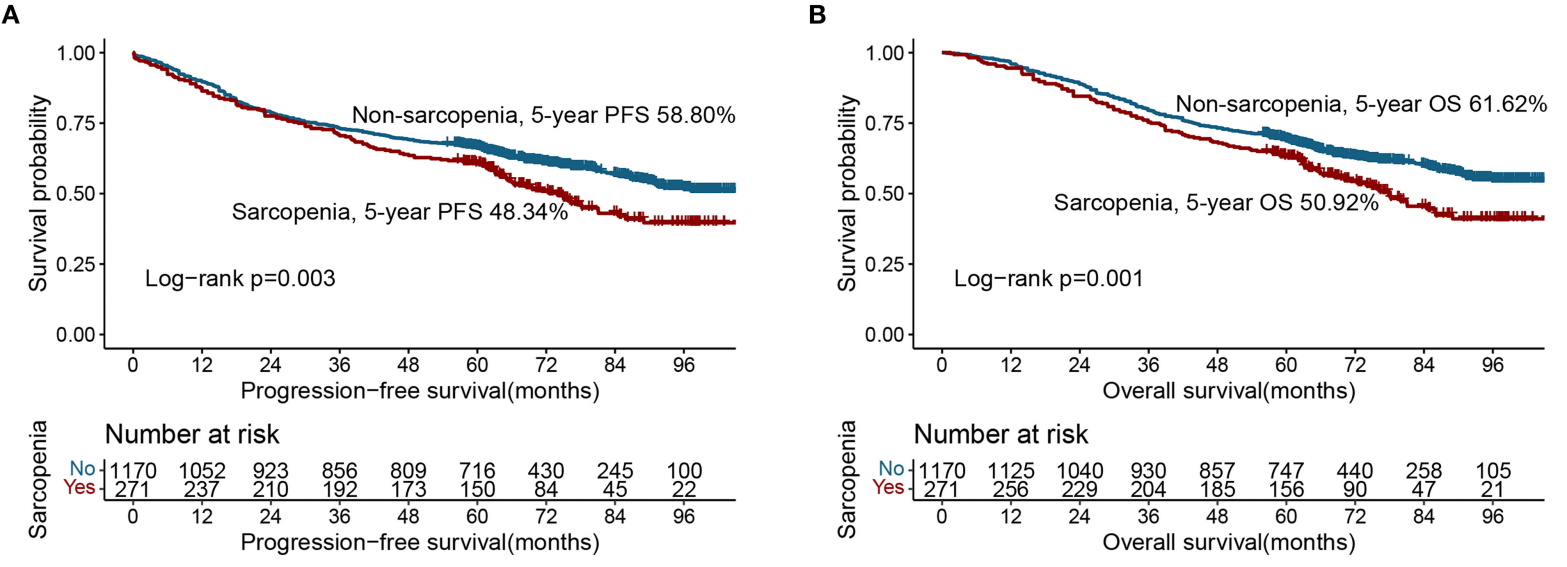
Kaplan-Meier curve of sarcopenia in CRC patients. **(A)** Progression-free survival; **(B)** Overall survival.

**Figure 2:**
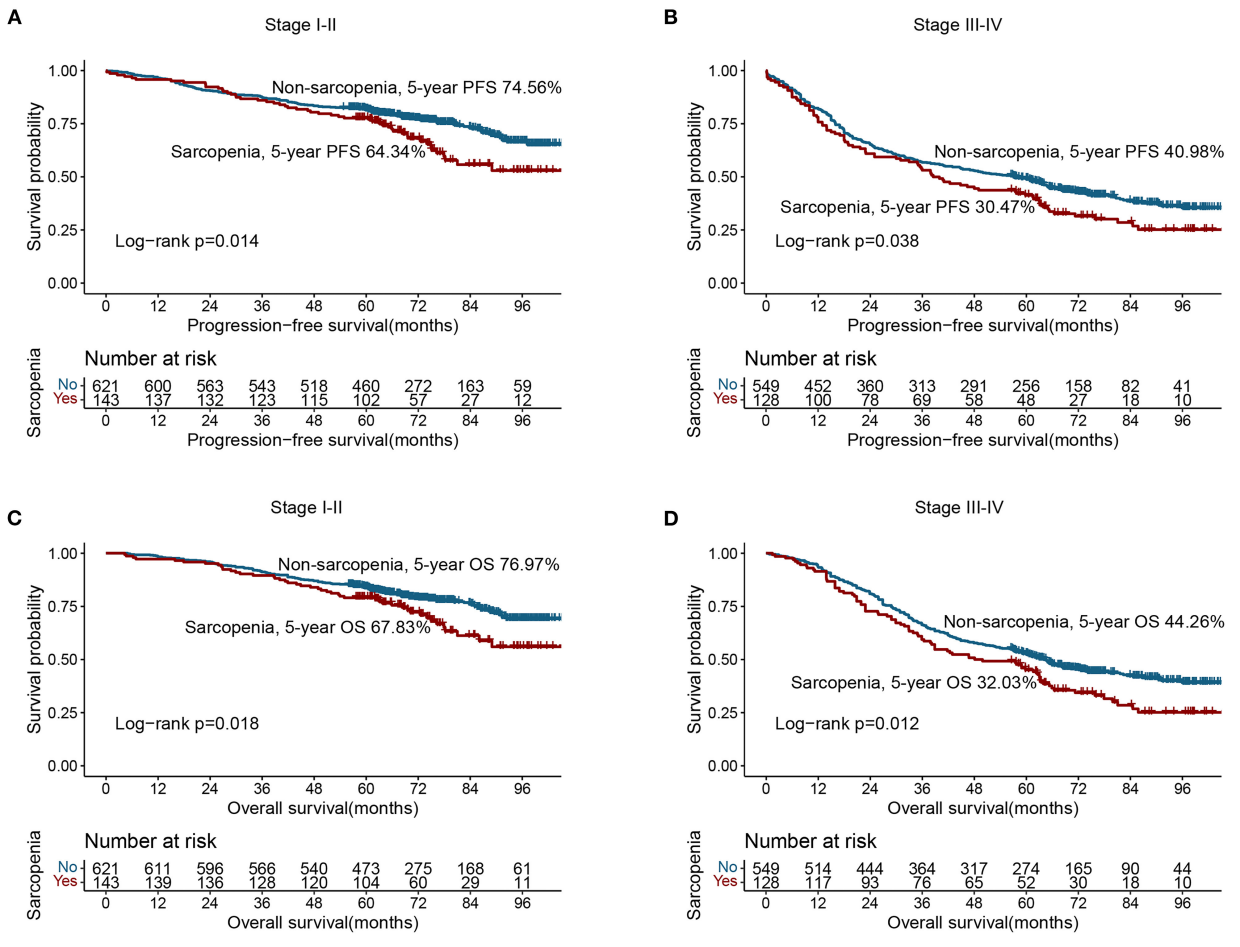
Stratified survival analysis of sarcopenia based on different TNM stage. **(A)** Progression-free survival of stage I–II; **(B)** Progression-free survival of stage III–IV; **(C)** Overall survival of stage I–II; **(D)** Overall survival of stage III–IV.

Based on the last follow-up, 400 patients had a recurrence, including 84 patients diagnosed with sarcopenia (31.0%) and 316 patients not diagnosed with sarcopenia (27.00%). The 5-year PFS of stage I-IV CRC patients were 78.52, 69.17, 47.04, and 7.30%, respectively. Compared with non-sarcopenia patients, the PFS of sarcopenia patients was worse (5-year PFS, 48.34 vs. 58.80%, *p* = 0.003) (Figure 1A). In the normal CEA subgroup, patients with sarcopenia had a significantly lower PFS than those without sarcopenia (Supplementary Figure S1A). However, no significant difference was observed in the high-CEA group (Supplementary Figure S1C). Notably, sarcopenia can provide effective prognostic stratification for CRC patients at different pathological stages (Figures 2A, B). In the univariate survival analysis, sarcopenia was associated with poor PFS in CRC patients (HR, 1.329; 95%CI: 1.101–1.605, *p* = 0.003). Multivariate survival analysis showed that, compared with non-sarcopenia patients, sarcopenia patients had a 23.9% higher risk of adverse PFS (HR, 1.239; 95%CI: 1.019–1.505, *p* = 0.031) (Table 2). Subgroup multivariate survival analysis showed that sarcopenia was an independent risk factor for PFS in most subgroups of CRC patients (Supplementary Figure S2A).

**Table 2:** Univariate and multivariate Cox regression analysis of clinicopathological characteristics associated with disease-free survival in CRC patients.

**Clinicopathological characteristics**	**Progression-free survival**			
	**Univariate analysis**		**Multivariate analysis**	
	**HR (95%CI)**	***P*** **-value**	**HR (95%CI)**	***P*** **-value**
Age	1.280 (1.093–1.499)	0.002	1.287 (1.091–1.519)	0.003
***T*** **stage**				
T1	Ref.			
T2	1.058 (0.564–1.986)	0.860	0.955 (0.506–1.800)	0.886
T3	2.281 (1.251–4.157)	0.007	1.337 (0.722–2.473)	0.355
T4	3.085 (1.676–5.679)	< 0.001	1.479 (0.789–2.772)	0.222
***N*** **stage**		< 0.001		
N0	Ref.			
N1	1.872 (1.553–2.257)	< 0.001	1.519 (1.251–1.844)	< 0.001
N2	4.055 (3.338–4.927)	< 0.001	2.754 (2.223–3.412)	< 0.001
*M* stage	5.384 (4.411–6.572)	< 0.001	3.292 (2.661–4.071)	< 0.001
Perineural invasion (Positive)	1.951 (1.584–2.403)	< 0.001	1.077 (0.839–1.381)	0.561
Vascular invasion (Positive)	1.755 (1.403–2.195)	< 0.001	1.259 (1.019–1.556)	0.033
**Pathological type**				
Protrude type				
Infiltrating type	1.466 (1.075–1.998)	0.016	1.258 (0.918–1.723)	0.153
Ulcerative type	1.366 (1.129–1.653)	0.001	1.143 (0.939–1.393)	0.183
Differentiation (High–medium)	0.700 (0.563–0.869)	0.001	0.847 (0.676–1.060)	0.147
Size (≥5 cm)	1.192 (1.019–1.395)	0.028	0.983 (0.834–1.158)	0.835
CEA (≥5 ng/ml)	1.988 (1.698–2.328)	< 0.001	1.484 (1.254–1.757)	< 0.001
Sarcopenia (Yes)	1.329 (1.101–1.605)	0.003	1.239 (1.019–1.505)	0.031

### 3.3. The relationship between sarcopenia and OS

A total of 582 patients died, including 133 (49.08%) with sarcopenia and 449 (38.38%) without sarcopenia. The 5-year OS of stage I-IV CRC patients were 81.34, 71.67, 50.37, and 8.76%, respectively. The Kaplan-Meier curve showed that the OS of patients with sarcopenia was significantly lower than that of patients without sarcopenia (5-year OS, 50.92 vs. 61.62%, *p* = 0.001) (Figure 1B). In the early stages of CRC (TNM stage I-II), the OS of patients with sarcopenia was significantly poorer than that of patients without sarcopenia (Figure 2C). In the advanced stage, sarcopenia was still effective in the prognostic differentiation of CRC patients (Figure 2D). Additionally, the subgroup survival analysis showed that sarcopenia could effectively differentiate the OS of CRC patients in the normal CEA subgroup (Supplementary Figure S1B). However, it could not significantly differentiate the prognosis of patients in the high CEA subgroup (Supplementary Figure S1D). Multivariate adjustment survival analysis showed that sarcopenia was independently associated with poor OS (HR: 1.273, 95%CI: 1.042–1.556, *p* < 0.001) in CRC patients (Table 3). Sarcopenia was subsequently discovered as an independent risk factor for OS in most subgroups (Supplementary Figure S2B).

**Table 3:** Univariate and multivariate Cox regression analysis of clinicopathological characteristics associated with overall survival in CRC patients.

**Clinicopathological characteristics**	**Overall survival**			
	**Univariate analysis**		**Multivariate analysis**	
	**HR (95%CI)**	***P*** **-value**	**HR (95%CI)**	***P*** **-value**
Age	1.344 (1.141–1.583)	< 0.001	1.315 (1.108–1.561)	0.002
***T*** **stage**				
T1	Ref.			
T2	1.008 (0.520–1.954)	0.981	0.868 (0.446–1.691)	0.678
T3	2.293 (1.223–4.302)	0.010	1.264 (0.663–2.409)	0.477
T4	3.162 (1.669–5.991)	< 0.001	1.396 (0.723–2.697)	0.320
***N*** **stage**		< 0.001		
N0	Ref.			
N1	1.875 (1.544–2.277)	< 0.001	1.511 (1.235–1.848)	< 0.001
N2	4.079 (3.339–4.983)	< 0.001	2.624 (2.103–3.275)	< 0.001
*M* stage	5.609 (4.581–6.866)	< 0.001	3.411 (2.748–4.234)	< 0.001
Perineural invasion (Positive)	1.713 (1.359–2.159)	< 0.001	1.018 (0.786–1.319)	0.890
Vascular invasion (Positive)	2.039 (1.691–2.459)	< 0.001	1.317 (1.060–1.637)	0.013
**Pathological type**				
Protrude type				
Infiltrating type	1.448 (1.050–1.997)	0.024	1.225 (0.884–1.698)	0.223
Ulcerative type	1.366 (1.120–1.665)	0.002	1.148 (0.935–1.408)	0.188
Differentiation (High–medium)	0.648 (0.521–0.807)	< 0.001	0.768 (0.611–0.965)	0.023
Size (≥5 cm)	1.308 (1.112–1.539)	0.001	1.096 (0.925–1.298)	0.289
CEA (≥5 ng/ml)	2.036 (1.730–2.397)	< 0.001	1.483 (1.246–1.767)	< 0.001
Sarcopenia (Yes)	1.374 (1.132–1.667)	0.001	1.273 (1.042–1.556)	0.018

### 3.4. Prognostic nomograms

**Figure 3:**
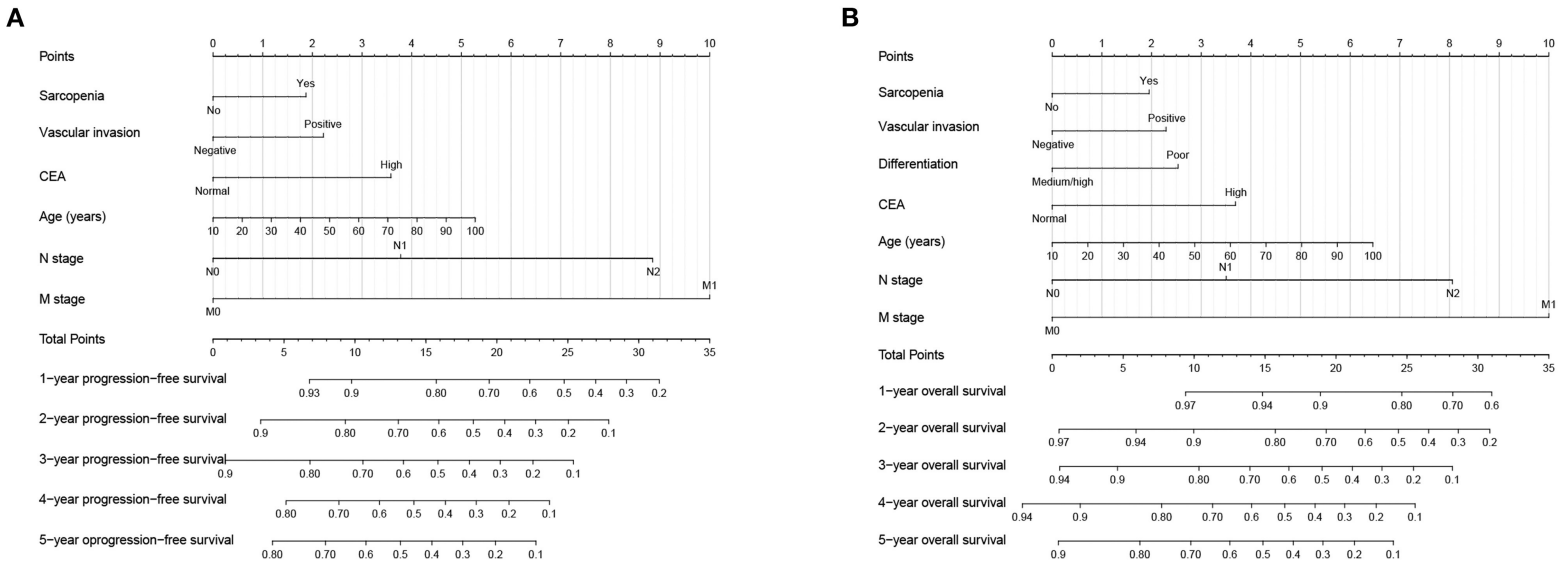
Construction prognostic nomograms in CRC patients. **(A)** The Progression-free survival nomogram; **(B)** The overall survival nomogram.

In the multivariate survival analysis, sarcopenia, vascular invasion, CEA, age, *N* stage, and *M* stage were independent factors affecting PFS in CRC patients. Therefore, we used these features to build a PFS nomogram to predict the 1–5 years of PFS in CRC patients (Figure 3A). The PFS nomogram showed that with the increase in CEA, the appearance of vascular invasion, the progress of *N* stage and *M* stage, the increase in age, and the emergence of sarcopenia, the predictive score increased, indicating that the risk of adverse PFS also increased. The C-index of the PFS nomogram was 0.717 (95%CI: 0.696–0.738). In addition, the calibration curves of the PFS nomogram at 3 and 5 years showed consistency between the predicted survival probability and the actual observation value (Supplementary Figures S3A, B).

Similarly, in the multivariate survival analysis of OS, sarcopenia, vascular invasion, differentiation, CEA, age, *N* stage, and *M* stage were all independently associated with OS in CRC patients. Therefore, the OS nomogram, which incorporates these clinicopathological features, was built to facilitate predicting the 1–5 years of OS in CRC patients (Figure 3B). The C-index of the OS nomogram was 0.724 (95%CI: 0.702–0.746). In addition, the calibration curves of the 3- and 5-year OS showed that the nomogram had good prediction accuracy (Supplementary Figures S3C, D).

In addition, according to a ratio of 7:3, we divided the total population into validation cohorts A (1,009) and B (432) for internal validation (Supplementary Table S1). The C-Index of PFS nomogram in validation cohort A and B was 0.710 (95% CI: 0.686–0.735) and 0.737 (95% CI: 0.701–0.773), respectively. In the OS nomogram, the C-index in validation cohorts A and B were 0.721 (95%CI: 0.696–0.746) and 0.733 (95%CI: 0.695–0.771), respectively. Calibration curves for the 3- and 5-year PFS and OS showed consistency between the predicted survival probability and the actual observation value in both validation A (Supplementary Figure S4A) and validation B (Supplementary Figure S4B).

## 5. Discussion

Sarcopenia is associated with poor clinical outcomes, including increased postoperative complications, postoperative hospitalization duration, increased inpatient costs, and even increased mortality (5, 6, 17–19). A Korean retrospective analysis showed that sarcopenia was associated with poor prognosis and increased chemotherapy toxicity in patients with stage III CRC who received preoperative neoadjuvant therapy (20). Nakanishi et al. (21) found that sarcopenia was significantly associated with disease progression and was an independent risk factor for poor outcomes in CRC patients. Malietzis et al. (22) retrospectively analyzed 805 CRC patients undergoing surgery and found that sarcopenia increased the risk of recurrence by 1.5 times and the risk of death by 1.7 times. Feliciano et al. (4) found that systemic inflammation was associated with sarcopenia in CRC patients. In addition, sarcopenia combined with systemic inflammation almost doubled the risk of death in CRC patients. The above evidence suggests that early screening, diagnosis, and intervention for sarcopenia in CRC patients have important clinical significance.

There are several ways to assess skeletal muscle mass. CT and MRI are more direct measurements of body composition. Their reliability has become the gold standard for estimating skeletal muscle mass (23). DXA is an accurate, reproducible, and widely used imaging modality for assessing body composition and is a commonly used radiological tool for diagnosing sarcopenia in clinical practice (9, 24). However, these measurement methods are limited in application because of the different degrees of radioactive radiation, high inspection costs, increased staffing, measurement tools and software, and operational complexity. BIA is a noninvasive, safe, simple, and radiation-free muscle mass determination method, but its accuracy has recently been questioned (25, 26). Recently, the newly developed anthropometric equation of skeletal muscle mass has gradually attracted much attention (12).

In this study, we found that sarcopenia, diagnosed based on anthropometric equations, is an independent risk factor for PFS and OS in CRC patients. Compared with patients without sarcopenia, those with sarcopenia had a 23.9 and 27.3% higher risk of developing poor PFS and OS, respectively. Sarcopenia also provides an excellent prognostic stratification for CRC patients with normal CEA levels. Moreover, sarcopenia was significantly associated with poor prognosis in most subgroups of CRC patients. TNM staging is an important tool for evaluating the prognosis of CRC patients, but the prognosis of patients with the same pathological stage is still different. We found that sarcopenia can effectively differentiate prognosis for patients with the same pathological stage; this indicates that additional assessment of sarcopenia can provide a more refined and accurate prognostic assessment for CRC patients.

In the correlation analysis, we noticed that older CRC patients were more likely to develop sarcopenia than younger CRC patients. Related studies also reported that the incidence of sarcopenia gradually increases with age; CRC patients older than 70 years have a sarcopenic incidence as high as 50% (27). With the aging population worldwide, sarcopenia has become an increasingly important issue that endangers public health (28). In addition, we found that CRC patients with tumor size >5 cm were more likely to develop sarcopenia. Patients with a large tumor burden were more likely to have complications, such as intestinal obstruction and bleeding, resulting in an increased risk of malnutrition. In addition, patients with a large tumor burden were often staged late and were in a state of high catabolism, low synthesis, and high inflammation. As a result, these patients have a significantly increased risk of developing sarcopenia. Overall, sarcopenia is both a phenotype that reflects disease progression and a predictor of adverse long-term outcomes in CRC patients.

Previous studies have shown that the 5-year OS of CRC patients was significantly different among stages. Research by Kittrongsiri et al. (29) showed the 5-year OS of stage I–IV CRC patients were 79.67, 67.50, 44.77, and 11.02%, respectively. Mangone et al. (30) found that the 5-year survival of colon cancer was 96.7, 83.4, 70.8, and 16.2%. In our study, the 5-year PFS/OS of stage I–IV CRC patients were (PFS: 78.52, 69.17, 47.04, and 7.30%; OS: 81.34, 71.67, 50.37, and 8.76%). The 5-year survival of CRC patients in this study was in the normal range. The differences in these studies may be affected by the heterogeneity of other pathological factors (such as histological types and differentiation, etc.), as well as the treatment level in different institutions and different periods.

We developed personalized prognostic nomograms to predict the 1–5 years of PFS/OS and OS in CRC patients. These prognostic nomograms integrated general information, sarcopenia, and pathological information and effectively evaluated the prognostic risk of CRC patients. The C-index and calibration curves showed that these nomograms had good prognostic accuracy. Subsequent internal validation cohorts confirmed the application value of these nomograms. These results indicate that our nomogram has wide application potential.

This study had some limitations. First, it adopted a cross-sectional retrospective analysis and failed to conduct multiple monitoring and evaluations. Second, this was a single-center study, and the sample size was relatively limited. In addition, the method for diagnosing sarcopenia was too single, and other skeletal muscle measurement methods were lacking in this study. Multicenter prospective studies are needed in the future to address these issues. Finally, the anthropometric equation also has certain limitations, because it is calculated based on algorithms. Although it has been verified by many queues, there may still be some heterogeneity due to individual differences. However, compared with CT, DXA and BIA, it does not require special equipment, does not cause radiation damage, is simple to calculate, and has strong operability, so it has a wide range of application prospects, suitable for popular grass-roots applications.

## 6. Conclusion

Sarcopenia diagnosed based on anthropometric equations is an independent risk factor for PFS and OS in CRC patients. The prognostic nomogram based on sarcopenia can provide a personalized reference for prognostic judgment and clinical decision-making of CRC patients.

## Data availability statement

The original contributions presented in the study are included in the article/Supplementary material, further inquiries can be directed to the corresponding authors.

## Ethics statement

The studies involving human participants were reviewed and approved by the Ethics Committee of the First Affiliated Hospital of Guangxi Medical University, with the approval number: 2021 (KY-E-043). The patients/participants provided their written informed consent to participate in this study.

## Author contributions

JG conception and design. JG and ST management support. SG, ML, YL, GY, QW, and YX data collection. HX data analysis and professional drafting. HX and LW manuscript writing. All authors contributed to the article and approved the submitted version.
