# Supplementary material for: Prognostic significance of sarcopenia diagnosed based on the anthropometric equation for progression-free survival and overall survival in patients with colorectal cancer
Source: Front Nutr. 2023 Feb 1;10:1076589. doi: 10.3389/fnut.2023.1076589 (PMC9928878; doi:10.3389/fnut.2023.1076589)
Supplement: Supplementary file 1 [file Table_1.DOCX]

**Figure S1.** Stratified survival analysis based on CEA level.


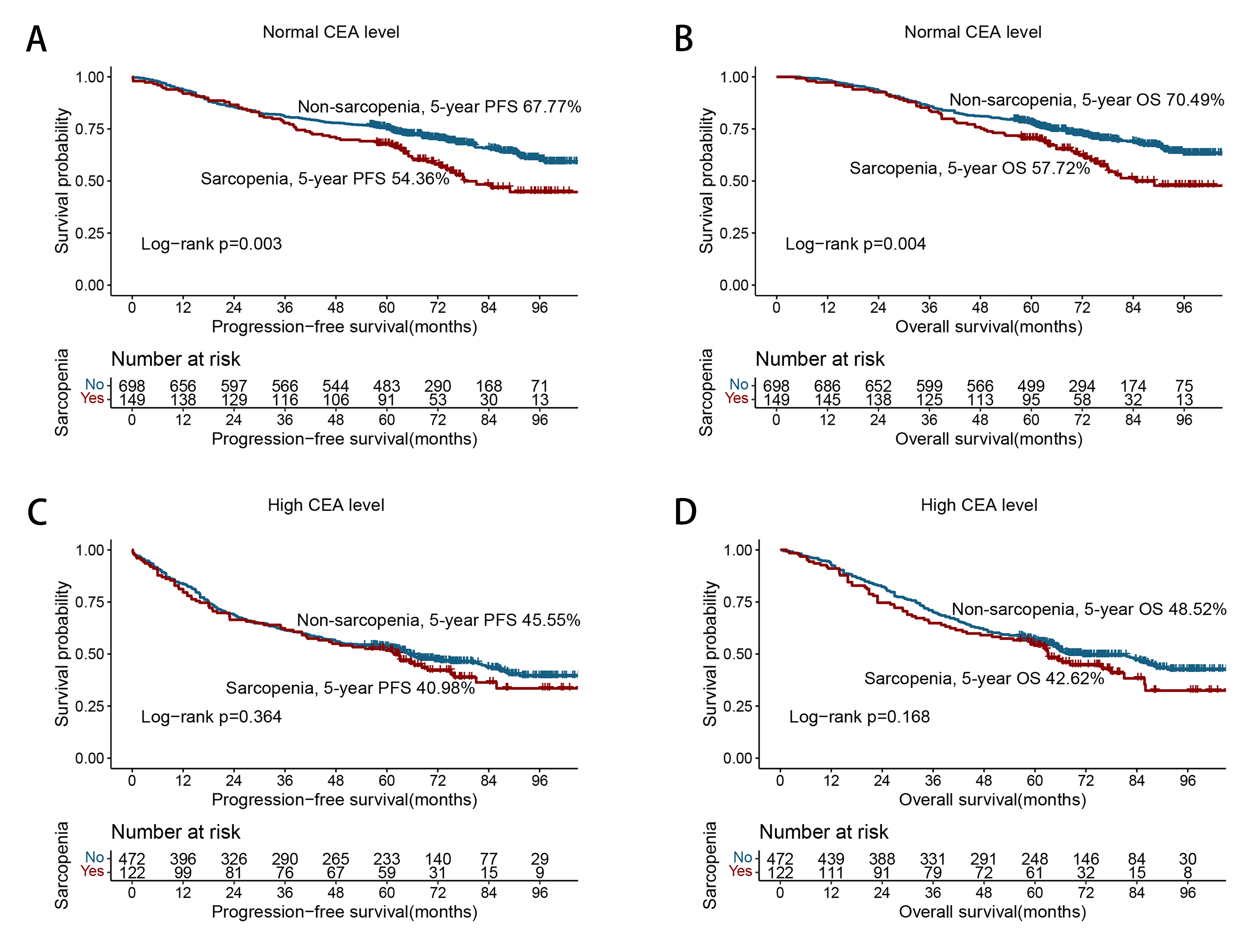


Notes: A, Progression-free survival of sarcopenia at normal CEA level; B, Overall survival of sarcopenia at normal CEA level; C, Progression-free survival of sarcopenia at high CEA level; D, Overall survival of sarcopenia at high CEA level.

**Figure S2.** The association between sarcopenia and hazard risk of survival in various subgroups. (A, Progression-free survival, B, Overall survival).


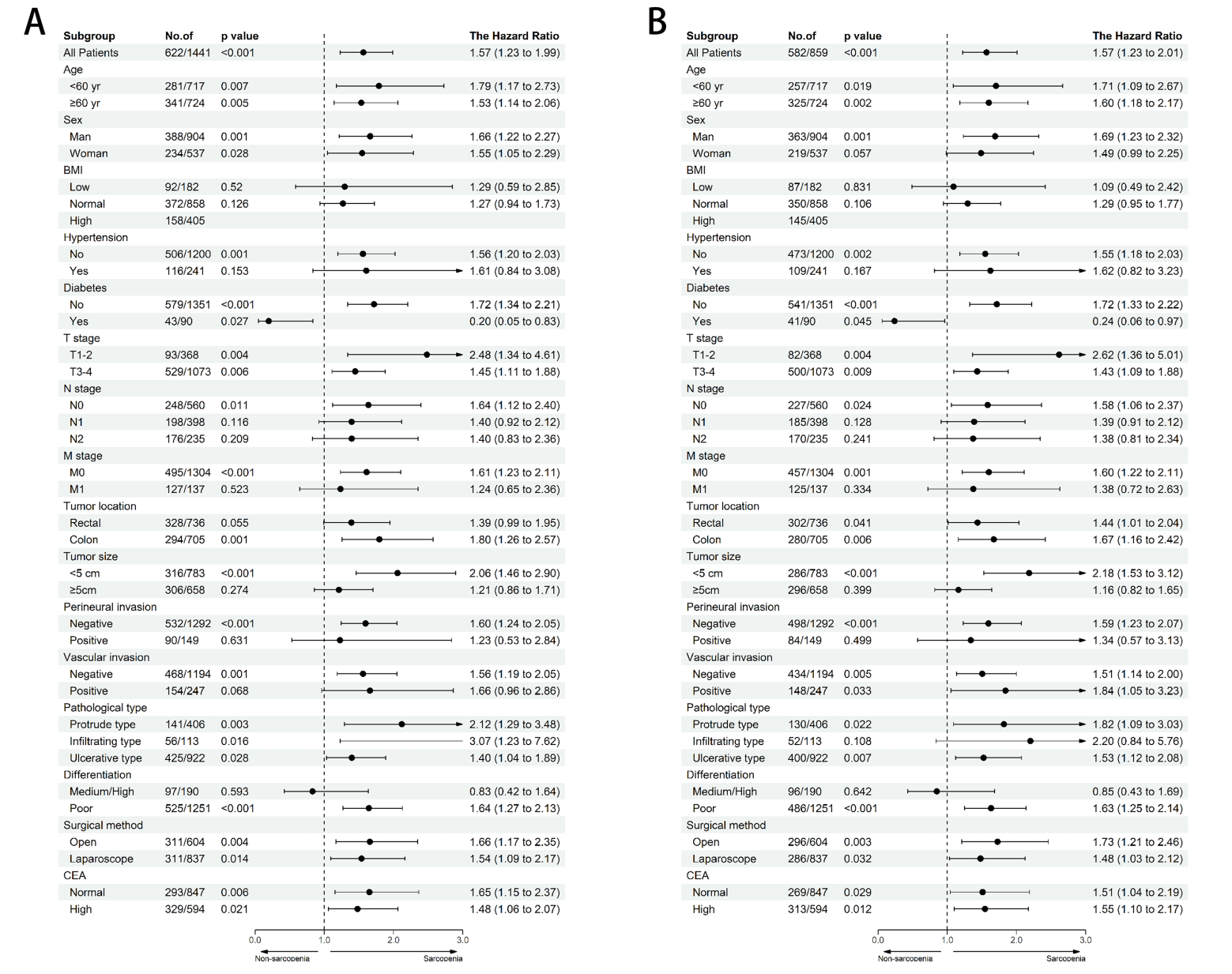


**Figure S3.** Calibration curve of the prognostic nomograms.

**
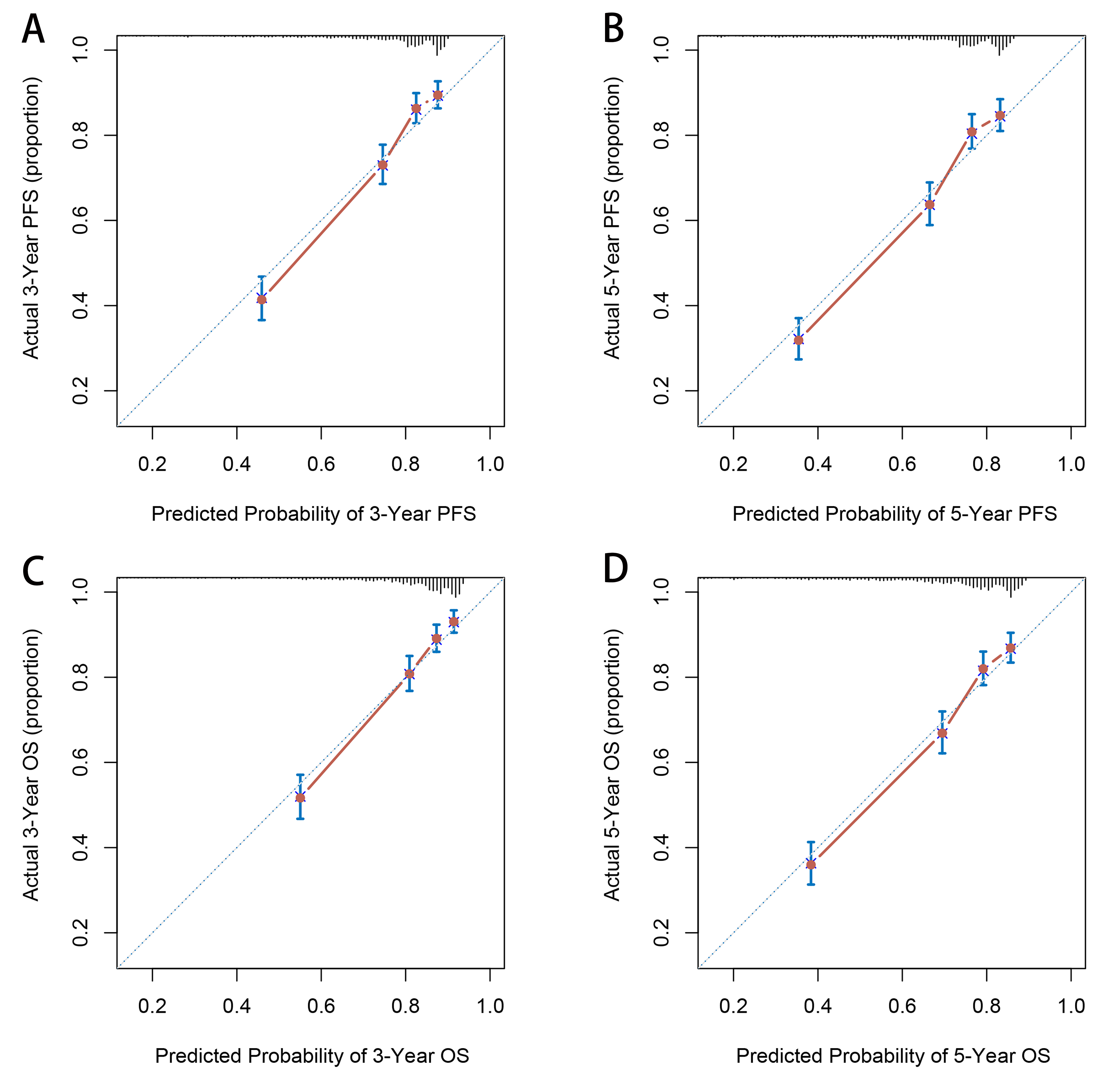
**

Notes: A, 3-year progression-free survival; B, 5-year progression-free survival; C, 3-year overall survival; D, 5-year overall survival.

**Figure S4.** Calibration curve at randomize internal validations.

**
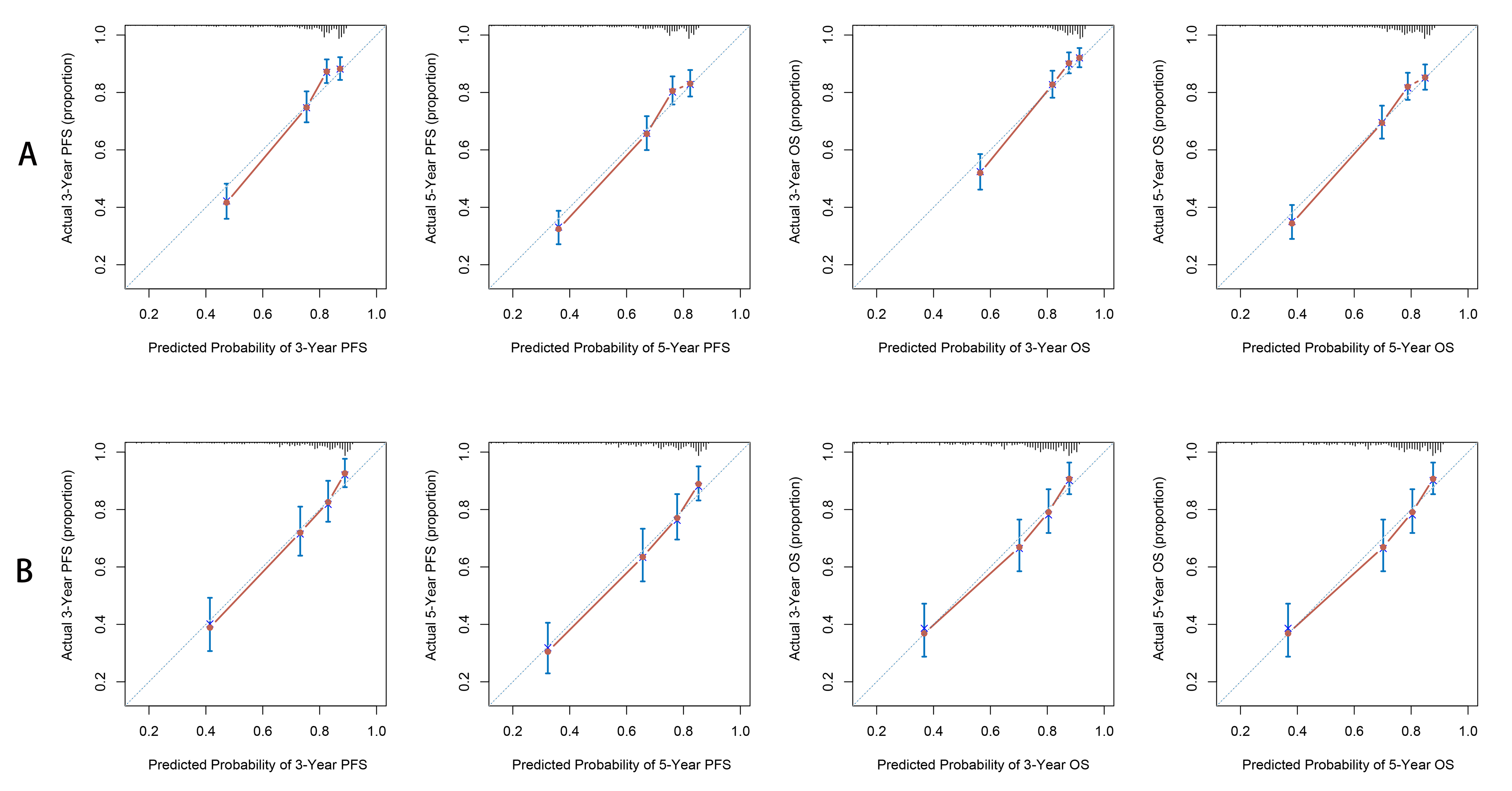
**

**Notes:** A, Validation cohort A; B, Validation cohort B.

**Table S1.** The clinicopathological characteristics of two validation cohorts in CRC patients.

| Clinicopathological characteristics | Validation a  (n = 1009) | Validation b  (n = 432) | P value |
| --- | --- | --- | --- |
| Sex(Man) | 641 (63.5) | 263 (60.9) | 0.372 |
| Age (mean (SD)) | 58.0 (13.2) | 58.4 (13.0) | 0.660 |
| BMI (median [IQR]) | 22.1 (20.0, 24.4) | 21.8 (19.9, 24.2) | 0.258 |
| Hypertension (Yes) | 172 (17.0) | 69 (16.0) | 0.672 |
| Diabetes (Yes) | 61 (6.0) | 29 (6.7) | 0.718 |
| T stage |  |  | 0.752 |
| T1 | 34 (3.4) | 16 (3.7) |  |
| T2 | 230 (22.8) | 88 (20.4) |  |
| T3 | 537 (53.2) | 233 (53.9) |  |
| T4 | 208 (20.6) | 95 (22.0) |  |
| N stage |  |  | 0.575 |
| N0 | 565 (56.0) | 243 (56.2) |  |
| N1 | 285 (28.2) | 113 (26.2) |  |
| N2 | 159 (15.8) | 76 (17.6) |  |
| M stage (Yes) | 89 ( 8.8) | 48 (11.1) | 0.208 |
| TNM stage (III-IV) | 477 (47.3) | 200 (46.3) | 0.777 |
| Perineural invasion (Yes) | 109 (10.8) | 40 ( 9.3) | 0.431 |
| Vascular invasion (Yes) | 188 (18.6) | 59 (13.7) | 0.026 |
| Macroscopic type | 276 (27.4) | 130 (30.1) | 0.393 |
| Protrude type | 84 (8.3) | 29 (6.7) |  |
| Infiltrating type | 649 (64.3) | 273 (63.2) |  |
| Ulcerative type | 867 (85.9) | 384 (88.9) | 0.150 |
| Differentiation (Poor) | 514 (50.9) | 222 (51.4) | 0.922 |
| Tumor location (Rectal) | 4.5 (3.5, 6.0) | 4.5 (3.5, 6.0) | 0.049 |
| Tumor size (median [IQR]) | 412 (40.8) | 182 (42.1) | 0.689 |
| CEA (High) | 417 (41.3) | 187 (43.3) | 0.527 |
| Surgical method (Open) | 97 (9.6) | 37 (8.6) | 0.597 |
| Radiotherapy (Yes) | 467 (46.3) | 190 (44.0) | 0.456 |
| Chemotherapy (Yes) | 405 (40.1) | 177 (41.0) | 0.813 |
| Death (Yes) | 17.0 (11.0, 21.0) | 17.0 (11.0, 21.0) | 0.891 |
| HOS (median [IQR]) | 49489.1 (44726.6, 55998.5) | 49627.9 (44088.7, 55963.3) | 0.634 |
| Hospitalization cost (median [IQR]) | 641 (63.5) | 263 (60.9) | 0.372 |

Table Note: CRC, colorectal cancer; BMI, body mass index.
